# A mutation in the *ZNF687* gene that is responsible for the severe form of Paget’s disease of bone causes severely altered bone remodeling and promotes hepatocellular carcinoma onset in a knock-in mouse model

**DOI:** 10.1038/s41413-023-00250-3

**Published:** 2023-03-14

**Authors:** Sharon Russo, Federica Scotto di Carlo, Antonio Maurizi, Giorgio Fortunato, Anna Teti, Danilo Licastro, Carmine Settembre, Tommaso Mello, Fernando Gianfrancesco

**Affiliations:** 1 Institute of Genetics and Biophysics “Adriano Buzzati-Traverso”, National Research Council of Italy, Naples, Italy; 2 Department of Environmental, Biological and Pharmaceutical Sciences and Technologies (DiSTABiF), University of Campania Luigi Vanvitelli, Caserta, Italy; 3 Department of Biotechnological and Applied Clinical Sciences, University of L’Aquila, L’Aquila, Italy; 4 AREA Science Park, Padriciano, Trieste, Italy; 5 Telethon Institute of Genetics and Medicine (TIGEM), Pozzuoli, Italy; 6 Gastroenterology Unit, Department of Experimental and Clinical Biochemical Sciences “Mario Serio”, University of Florence, Florence, Italy; 7 IRCCS INM Neuromed, Pozzilli, IS Italy

**Keywords:** Bone, Bone cancer

## Abstract

Paget’s disease (PDB) is a late-onset bone remodeling disorder with a broad spectrum of symptoms and complications. One of the most aggressive forms is caused by the P937R mutation in the *ZNF687* gene. Although the genetic involvement of *ZNF687* in PDB has been extensively studied, the molecular mechanisms underlying this association remain unclear. Here, we describe the first *Zfp687* knock-in mouse model and demonstrate that the mutation recapitulates the PDB phenotype, resulting in severely altered bone remodeling. Through microcomputed tomography analysis, we observed that 8-month-old mutant mice showed a mainly osteolytic phase, with a significant decrease in the trabecular bone volume affecting the femurs and the vertebrae. Conversely, osteoblast activity was deregulated, producing disorganized bone. Notably, this phenotype became pervasive in 16-month-old mice, where osteoblast function overtook bone resorption, as highlighted by the presence of woven bone in histological analyses, consistent with the PDB phenotype. Furthermore, we detected osteophytes and intervertebral disc degeneration, outlining for the first time the link between osteoarthritis and PDB in a PDB mouse model. RNA sequencing of wild-type and *Zfp687* knockout RAW264.7 cells identified a set of genes involved in osteoclastogenesis potentially regulated by Zfp687, e.g., *Tspan7*, *Cpe*, *Vegfc*, and *Ggt1*, confirming its role in this process. Strikingly, in this mouse model, the mutation was also associated with a high penetrance of hepatocellular carcinomas. Thus, this study established an essential role of Zfp687 in the regulation of bone remodeling, offering the potential to therapeutically treat PDB, and underlines the oncogenic potential of ZNF687.

## Introduction

Paget’s disease of bone (PDB) is a late-onset skeletal disorder characterized by impaired bone remodeling activity due to high bone degradation activity by osteoclasts followed by disorganized bone deposition by osteoblasts.^1,2^ PDB affects one (monostotic) or more (polyostotic) skeletal sites, and although any bone can be affected, there is a predilection for the skull, spine, pelvis, femur, and tibia. Bone pain, deformity, and pathological fractures, typically in the lytic phase of the disease, are the most common symptoms.^2^

A common complication of PDB is osteoarthritis (OA).^3^ Indeed, patients with PDB are more likely than age-matched controls to need total hip and knee arthroplasty as a result of secondary OA.^4^ A peculiar feature of OA is the formation of osteophytes, which are osteocartilaginous outgrowths that typically form at the joint margins in the region where the synovium attaches to the edge of the articular cartilage and merges with the periosteum.^5^ Osteophytes are established through the growth of an initial cartilage template that is at least partially replaced with bone-containing marrow cavities.^6^ In PDB, the osteophyte occurrence is less clear. The original description of Sir James Paget in 1876 included the postmortem analysis of a pathologic specimen of the femur of case 5, which demonstrated features of PDB at the proximal and distal femur, with femoral head remodeling and osteophyte formation.^7^ However, whether the formation of osteophytes is a prominent feature of PDB remains to be determined.

The most relevant PDB complication is the neoplastic degeneration of affected bones in osteosarcoma (OS/PDB) or giant cell tumor (GCT/PDB).^8^ Although rare, their occurrence, commonly observed in polyostotic PDB, is associated with more severe manifestations of the disease and reduced life expectancy. Osteosarcoma is the most severe malignant transformation of PDB and shows an extremely poor prognosis that has not improved over the years, showing a 5-year survival rate almost nil, especially after metastasis to the lungs.^9^ In contrast, GCT rarely metastasizes but is locally aggressive.^10,11^ In the last decade, it has been proven that the distinct forms of the disease have different genetic bases.^12–15^ The common form of PDB is due to mutations in the *SQSTM1* gene, encoding the p62 protein, which is involved in important cellular mechanisms, such as autophagy or the regulation of the NFκB signaling pathway.^12,13^ Mutations in *SQSTM1* lead to an impaired and continuously activated NFκB pathway, resulting in more activated and multinucleated osteoclasts.^16^ Two *Sqstm1* knock-in mouse models have been generated, harboring the most common mutation (P392L), where mutant mice developed osteolytic/osteosclerotic lesions predominantly affecting the long bones but not involving the spine.^17–19^ Interestingly, the most common antiresorptive treatment with bisphosphonates prevented the formation of pagetic-like lesions in mutant animals.^19^ PDB associated with giant cell tumor is instead negative for *SQSTM1* mutations and caused by a founder mutation (P937R) in the *ZNF687* gene. *ZNF687*-mutated patients present with a more severe clinical phenotype in terms of age of onset and number of affected sites.^14^ Importantly, unlike *SQSTM1* mutation carriers, *ZNF687*-mutated patients show an inadequate response to bisphosphonates and need massive doses of antiresorptive drugs to cure the disease.^20^ Moreover, pagetic patients harboring the *ZNF687* mutation were found to develop OA degeneration in 42.8% of cases as a PDB complication.^10,14^ However, the role of *ZNF687* in bone metabolism needs to be further explored. Although *ZNF687* seems to be under the transcriptional regulation of NFκB,^14^ the pathway in which it is involved appears to be different than *SQSTM1*. To elucidate the molecular and pathological role of *ZNF687* in PDB, in this study, we generated a knock-in mouse model carrying the P937R mutation in the homologous *Zfp687* gene and performed skeletal characterization.

## Results

### *ZNF687* is upregulated during human and murine osteoclastogenesis

**Fig. 1:**
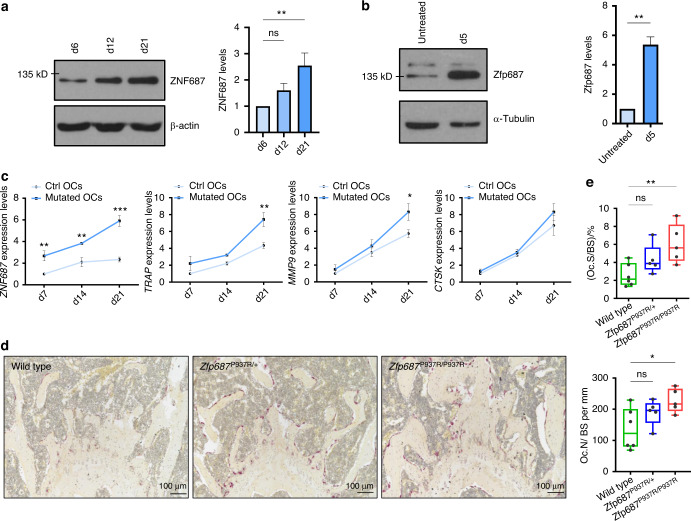
ZNF687 has a key role in osteoclastogenesis. **a** Western blot detection (left) and densitometric quantification (right) of ZNF687 protein levels in differentiated osteoclasts derived from a healthy donor upon sRANKL stimulation of PBMCs at days 6, 12, and 21. Western blots were normalized with β-actin. Data are presented as the mean ± s.d. Statistical significance was assessed by one-way ANOVA with Dunnett’s multiple comparison test (**P < 0.01). **b** Western blot detection (left) and densitometric quantification (right) of Zfp687 protein levels in differentiated osteoclasts derived from RAW264.7 cells subjected to sRANKL stimulation for 5 days. Western blots were normalized with α-tubulin. Data are presented as the mean ± s.d. Statistical significance was assessed by unpaired *t* test (**P < 0.01, two-tailed). **c** Bar graphs showing gene expression analysis of *ZNF687* and the osteoclastogenic markers *TRAP*, *MMP9*, and *CTSK* during osteoclast differentiation of healthy and P937R-mutated PBMCs at days 7, 14, and 21. Data are presented as the mean ± s.d. Statistical significance was assessed by two-way ANOVA with Sidak’s multiple comparison test (**P < 0.01; ***P < 0.001). **d** Representative TRAP-stained sections of femoral growth plates of 3-month-old wild-type, *Zfp687*^P937R/+^, and *Zfp687*^P937R/P937R^ mice showing osteoclastic activity in purple; nuclei were counterstained with hematoxylin. **e** Box plots showing the osteoclast surface to bone surface ratio (Oc.S/BS; top) and osteoclast number to bone surface ratio (Oc.N/BS; bottom) in *Zfp687*^P937R/+^ (*n* = 5) and *Zfp687*^P937R/P937R^ mice (*n* = 5) compared with wild-type mice (*n* = 6) at 3 months of age. Data are presented as the median ± s.d. Statistical significance was assessed by one-way ANOVA with Dunnett’s multiple comparison test (*P < 0.05)

Previously, we profiled the expression of *ZNF687* during the osteoclast differentiation of peripheral blood mononuclear cells (PBMCs) from healthy donors and noted a progressive upregulation of its expression.^14^ Here, we first confirmed this upregulation at the protein level during the physiological differentiation process in humans and mice, confirming the key role of ZNF687 (Fig. 1a, b). Interestingly, *ZNF687*-mutated osteoclasts showed a greater expression of *ZNF687* itself than those of healthy individuals, supporting the gain-of-function status of the P937R mutation (Fig. 1c, left). Additionally, the expression of genes whose upregulation is crucial for successful osteoclastic differentiation (e.g., *TRAP*, *MMP9*, *CTSK*) was increased in mutation-bearing osteoclasts (Fig. 1c, right). These data agree with a previous study demonstrating that patient-derived monocytes formed larger and more active osteoclasts in response to pro-osteoclastic stimuli in vitro.^20^ We therefore underline the relevance of ZNF687 in osteoclast biology, highlighting its positive modulatory effect on the osteoclastogenic process.

### *ZNF687* mutation alters bone cell differentiation processes

**Fig. 2:**
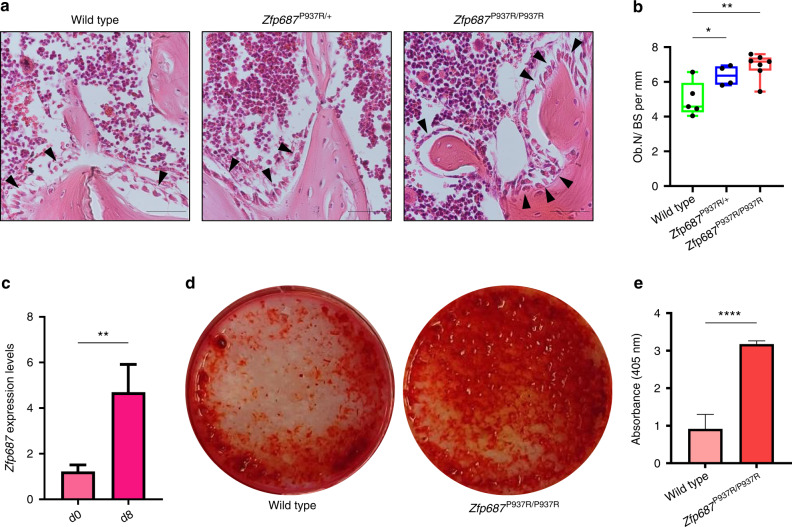
*Zfp687*^P937R^ differentiated osteoblasts display an increased mineralization potential. **a** Representative H&E-stained images of proximal tibial sections analyzed for osteoblast (arrowheads) quantification in wild-type, *Zfp687*^P937R/+^, and *Zfp687*^P937R/P937R^ mice at 3 months of age. Scale bars 50 µm. **b** Box plots showing histomorphometric quantification of the osteoblast number per bone surface ratio (Ob.N/BS) in wild-type (*n* = 5), *Zfp687*^P937R/+^ (*n* = 4), and *Zfp687*^P937R/P937R^ (*n* = 7) mice at 3 months of age. Data are presented as the median ± s.d. Statistical significance was assessed by one-way ANOVA with Dunnett’s multiple comparison test (*P < 0.05; **P < 0.01). **c** Bar graph showing *Zfp687* expression analysis in wild-type BM-MSCs either untreated (d0) or stimulated for 8 days toward osteoblastogenesis (d8). Data are presented as the mean ± s.d. Statistical significance was assessed by an unpaired *t* test (**P < 0.01, two-tailed). **d** Representative Alizarin Red Staining (ARS) images of wild-type and *Zfp687*^P937R/P937R^ BM-MSCs after 8 days of osteogenic induction. **e** Bar graph showing ARS quantification of wild-type (*n* = 4) and *Zfp687*^P937R/P937R^ (*n* = 4) osteoblast differentiation after 8 days of osteogenic induction. Data are presented as the mean ± s.d. Statistical significance was assessed by an unpaired *t* test (****P < 0.000 1, two-tailed)

To deepen the understanding of the effect of the *ZNF687* mutation on bone metabolism, we generated a knock-in mouse model harboring the c.2810C>G (P937R) mutation detected in GCT/PDB patients.^14,20^ The mutation has been introduced through site-directed mutagenesis in the targeting vector containing arms of the homologous *Zfp687* mouse gene, exploiting the 80.4% nucleotide homology between the human and mouse genes, even higher (84%) at exon 6, where the mutation is located (Fig. S1a, b). Both heterozygous and homozygous mice (referred to herein as *Zfp687*^P937R/+^ and *Zfp687*^P937R/P937R^, respectively) were viable and fertile, and the Mendelian distribution of genotypes in the litters was respected. Three-dimensional microcomputed tomography (µCT) evaluation of the trabecular bone composition of femurs and L4 lumbar vertebrae, as well as the cortical bone of the femoral midshaft of 3-month-old mice, was analyzed, revealing neither skeletal abnormalities nor bone volume alterations in mutant animals (Fig. S2, S3). Although there are no macroscopic differences at this stage, we observed different bone cellular activities. First, femur sections were analyzed for tartrate-resistant acid phosphatase (TRAP) expression, revealing increased osteoclast-dependent activity in mutant bones (Fig. 1d). Accordingly, we found significantly higher levels of both osteoclast surface (Oc.S/BS) and number (Oc.N/BS) per bone surface in histological sections of *Zfp687*^P937R/P937R^ mice, and a similar trend was observed in *Zfp687*^P937R/+^ mice (Fig. 1e). Interestingly, we also noted an increased osteoblast number (Ob.N/BS) per bone surface within histological sections of mutant mice, especially homozygous mice (Fig. 2a, b). Taken together, these results underline the positive effect of the P937R mutation on the number and activity of bone cells even before overt phenotypic manifestation. On the one hand, the effect of the *ZNF687* mutation on osteoclasts was previously explored;^14,20^ on the other hand, nothing is known about osteoblast differentiation. To fill this gap, we isolated bone marrow-derived mesenchymal stem cells (BM-MSCs) from 8-week-old wild-type and homozygous mutant mice, which were then subjected to osteoblast differentiation. We first demonstrated a strong upregulation of *Zfp687* during the physiological differentiation process, allowing us to hypothesize a role for this transcription factor in regulating osteoblast differentiation (Fig. 2c). In fact, *Zfp687*^P937R/P937R^ osteoblasts manifested a remarkably higher capability of mineralization and bone nodule formation in the presence of osteogenic factors (ascorbic acid and β-glycerophosphate) than wild-type osteoblasts (Fig. 2d). Notably, mutant cells became fully differentiated osteoblasts as early as 8 days after osteogenic induction, while wild-type cells displayed an expected slower differentiation process (Fig. 2d, e). We therefore conclude that the P937R mutation accelerates osteoblast formation and function, consistent with what was expected in bone remodeling alterations leading to PDB.

**Fig. 3:**
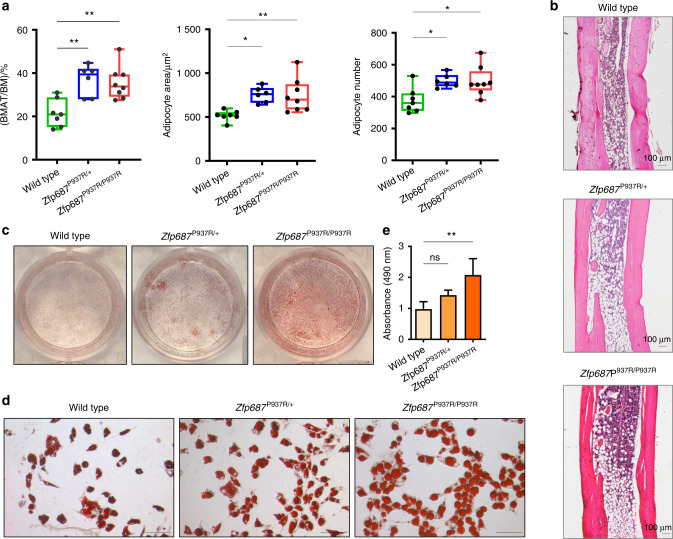
*Zfp687* mutant mice display increased cBMAT composition at 3 months of age. **a** Box plots showing the quantification of the tibial cBMAT volume expressed as the percentage of BMAT to total bone marrow ratio (BMAT/BM; left), the adipocyte area (middle), and the adipocyte number (right) of wild-type (*n* = 7), *Zfp687*^P937R/+^ (*n* = 6), and *Zfp687*^P937R/P937R^ (*n* = 8) mice at 3 months of age. Data are presented as the median ± s.d. Statistical significance was assessed by one-way ANOVA with Dunnett’s multiple comparison test (*P < 0.05; **P < 0.01). **b** Representative H&E-stained images of distal tibial sections analyzed for adipocyte measurements of the indicated genotypes. **c**, **d** Oil Red O (ORO) staining of wild-type, *Zfp687*^P937R/+^, and *Zfp687*^P937R/P937R^ BM-MSCs upon adipogenic induction and differentiation (plate view in **c**) and 20X magnification in (**d**). Scale bars, 100 µm. **e** Bar graphs show the intensity of ORO staining (absorbance at 490 nm) of wild-type (*n* = 4), *Zfp687*^P937R/+^ (*n* = 5), and *Zfp687*^P937R/P937R^ (*n* = 6) adipocytes. Data are presented as the mean ± s.d. Statistical significance was assessed by one-way ANOVA with Dunnett’s multiple comparison test (**P < 0.01)

During the histological analyses conducted on 3-month-old mice, we surprisingly observed an increase in marrow adipocytes in mutant femurs. A mutual correlation between bone marrow adipose tissue (BMAT) and bone loss exists;^21–24^ however, no research has been conducted to analyze this correlation in PDB. Therefore, we decided to quantify BMAT in the *Zfp687* mouse model. The distal tibiae of wild-type and mutant mice at 3 months of age were stained with hematoxylin-eosin and subjected to measurement of constitutive bone marrow adipose tissue (cBMAT). Our analysis highlighted that cBMAT was increased by ~1.5-fold in both *Zfp687*^P937R/+^ and *Zfp687*^P937R/P937R^ mice (*P* = 0.009 9 and *P* = 0.007 9) compared to wild-type mice (Fig. 3a, b). Both adipocyte number and area increased in mutant sections (Fig. 3a), indicating that mutant bone marrow contains more and larger fat cells. To confirm this observation, we subjected BM-MSCs to adipocyte differentiation in vitro, and as expected, mutant adipocytes appeared larger than wild-type cells and contained a large amount of lipid droplets, as shown by ORO staining (Fig. 3c–e).

Taken together, these data reveal that, in addition to the osteoclast differentiation program, stromal cell commitment is also altered by the mutation in *Zfp687*.

### Adult *Zfp687* mutant mice show trabecular bone loss and initial altered bone deposition

**Fig. 4:**
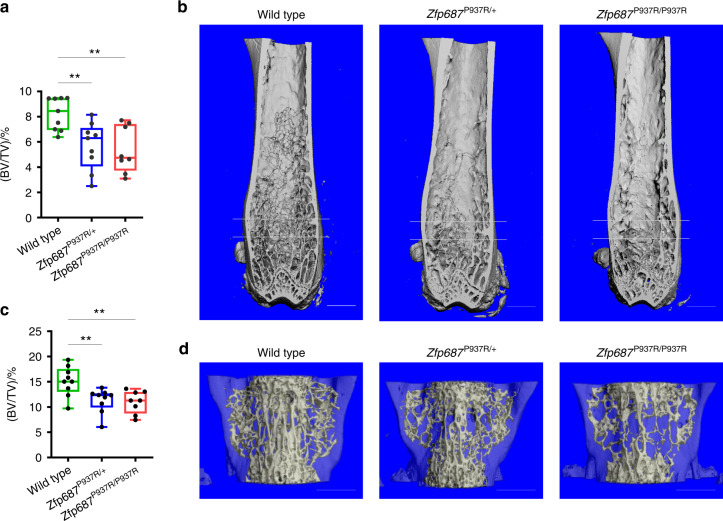
*Zfp687* mutant mice show remarkable trabecular bone loss at the appendicular and axial skeleton at 8 months of age. **a** Box plots showing the percentage of BV/TV by µCT in the trabecular bone of the femoral distal epiphysis of 8-month-old wild-type (*n* = 9), *Zfp687*^P937R/+^ (*n* = 9), and *Zfp687*^P937R/P937R^ (*n* = 8) mice. The region between the two gray lines represents the region of interest selected for the trabecular analysis. Data are presented as the median ± s.d. Statistical significance was assessed by one-way ANOVA with Dunnett’s multiple comparison test (**P < 0.01). **b** Representative µCT 3D reconstruction showing trabecular bone of femurs from wild-type, *Zfp687*^P937R/+^, and *Zfp687*^P937R/P937R^ mice. Scale bars 1 mm. **c** Box plots showing the percentage of BV/TV by µCT in the trabecular bone of the L4 vertebra of 8-month-old wild-type (*n* = 9), *Zfp687*^P937R/+^ (*n* = 9), and *Zfp687*^P937R/P937R^ (*n* = 8) mice. Data are presented as the median ± s.d. Statistical significance was assessed by one-way ANOVA with Dunnett’s multiple comparison test (**P < 0.01). **d** Representative µCT 3D reconstruction of L4 vertebrae from wild-type, *Zfp687*^P937R/+^, and *Zfp687*^P937R/P937R^ mice. Scale bars 1 mm

**Fig. 5:**
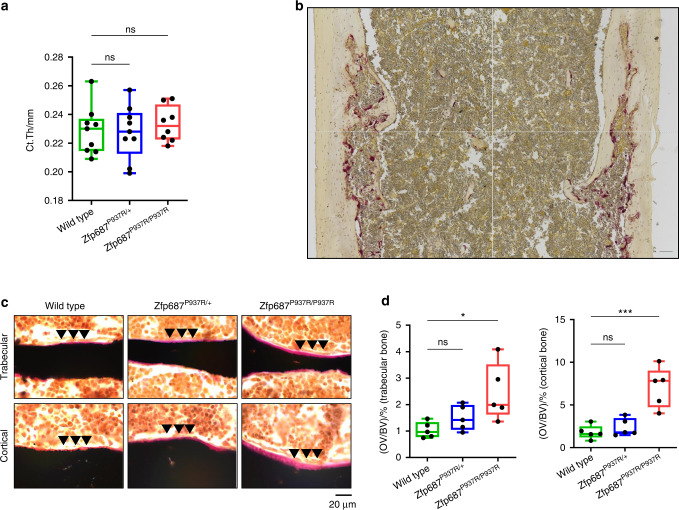
Severe impairment of bone remodeling in *Zfp687* mutant mice at 8 months of age. **a** Box plots showing quantitative measurements of cortical thickness of the femoral midshaft by µCT of 8-month-old wild-type (*n* = 9), *Zfp687*^P937R/+^ (*n* = 9), and *Zfp687*^P937R/P937R^ (*n* = 8) mice. Data are presented as the median ± s.d. Statistical analysis was assessed by one-way ANOVA with Dunnett’s multiple comparison test. **b** TRAP staining of a femur section of a *Zfp687* mutant mouse depicting a representative cortical osteolytic lesion. The figure is the result of a mosaic of 4 images of adjacent regions taken at 10X magnification. Scale bar, 100 µm. **c** Representative von Kossa and van Gieson images of trabecular (upper) and cortical (lower) femur sections of wild-type, *Zfp687*^P937R/+^, and *Zfp687*^P937R/P937R^ mice. Mineralized bone (black) and osteoid (pink) are visualized. Arrowheads indicate increased osteoid deposition. **d** Box plots showing quantification of osteoid volume over bone volume percentage (OV/BV) from von Kossa-stained sections counterstained with van Gieson in trabecular (left) and cortical (right) bone of wild type (*n* = 5), *Zfp687*^P937R/+^ (*n* = 5), and *Zfp687*^P937R/P937R^ (*n* = 5) mice. Data are shown as the median ± s.d. Statistical significance was assessed by one-way ANOVA with Dunnett’s multiple comparison test (*P < 0.05; **P < 0.01; ***P < 0.001)

To further elucidate the early phase of Paget’s disease, we subjected mice to skeletal phenotyping at 8 months of age, which corresponds to approximately 30 years in humans, considering that the initial PDB diagnosis in *ZNF687*-mutated patients is generally at approximately 45 years of age.^14^ Parametric analysis revealed significant bone mass reduction affecting the hind limbs and spine of mutant mice. In particular, the ratio of bone volume (BV) to total volume (TV; BV/TV) of femoral trabecular bone was decreased by 31% in *Zfp687*^P937R/+^ and 35% in *Zfp687*^P937R/P937R^ mice compared to wild-type mice (*P* = 0.007 and *P* = 0.003, respectively) (Fig. 4a, b). The bone trabecular mass reduction was mainly due to a decrease in trabecular number (Tb.N) and, consequently, an increase in trabecular separation (Tb.Sp) (Fig. S4a). Additionally, trabecular bone mass in the spine was reduced by 25% (*P* = 0.008) and 27% (*P* = 0.005) in the L4 vertebrae of *Zfp687*^P937R/+^ and *Zfp687*^P937R/P937R^ mice, respectively, with sparser and thinner trabeculae than wild-type littermates (Figs. 4c, d, S4b). Although significant cortical thickening (Fig. 5a), bone expansion, or deformity was not found at the midshaft of femurs of mutant animals, we sporadically observed the presence of lytic lesions affecting the cortical bone of long bones, in which TRAP-positive osteoclasts appeared giant-sized and multinucleated compared to the controls (Fig. 5b). A closer look at the osteoblast activity of 8-month-old mutant mice revealed that dysregulated bone deposition also occurred. In fact, histomorphometric analysis of femur sections through von Kossa/van Gieson staining showed an increased osteoid volume at both the trabecular and cortical levels in *Zfp687*^P937R/P937R^ mice that was compatible with an enhanced deposition of non-mineralized bone matrix (Fig. 5c, d). To examine whether this increased osteoid deposition was due to a generalized mineralization defect, we repeated von Kossa/van Gieson staining in younger mice (3 months old). We did not detect any increase in osteoid thickness in the mutant samples compared with the WT samples (data not shown), indicating that altered mineralization only occurred in adult mice. Collectively, these data indicate that the P937R mutation leads to bone mass reduction and altered matrix deposition in 8-month-old mice.

### *Zfp687* mutation causes a severe PDB-like phenotype in aged mice

**Fig. 6:**
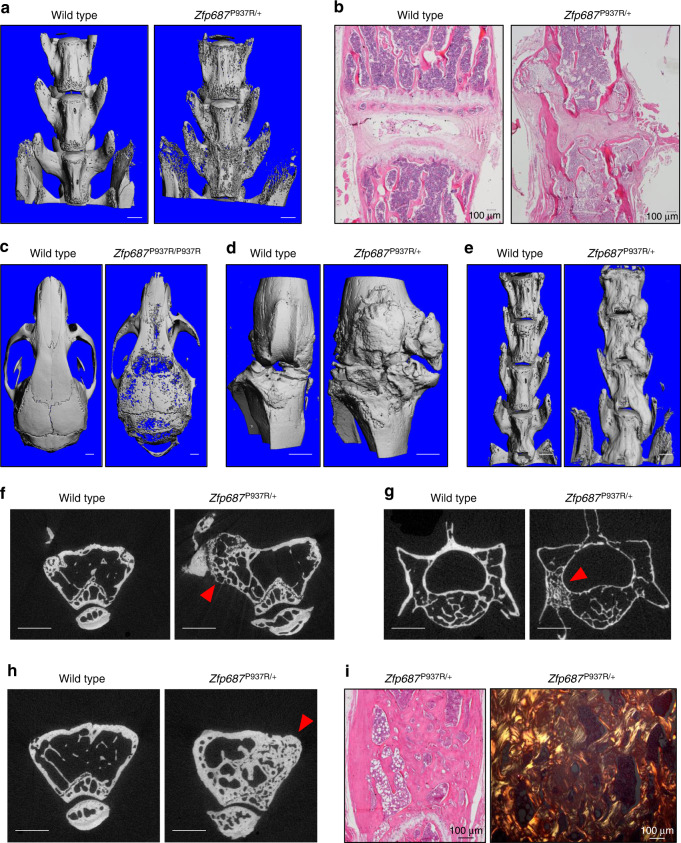
Aged *Zfp687*^P937R^ mutant mice develop pagetic lesions and osteophytes. **a** Representative µCT reconstructed 3D images of the spine (lumbar vertebrae) of wild-type and *Zfp687*^P937R/+^ mice, showing osteolytic cortical lesions and vertebral fusion in mutant mice at 16 months. **b** Representative H&E-stained sections showing the intervertebral disk degeneration and cartilage degradation of the joint space between lumbar vertebrae of a *Zfp687*^P937R/+^ mutant animal compared to a wild type animal (left). **c** Representative µCT 3D images of osteolytic cortical lesions in calvarial bone of the *Zfp687*^P937R/+^ mutant (right) compared to the wild type (left). Scale bar 1 mm. **d**, **e** Representative µCT 3D images of osteophyte formation at the knee joint (in **d**) and lumbar vertebrae (in **e**) of wild-type and *Zfp687*^P937R^ mutant mice. Scale bar 1 mm. **f** Representative µCT cross-sections showing microarchitectural changes in subchondral bone (arrowhead) corresponding to osteophyte formation in the femur of the *Zfp687*^P937R/+^ mutant compared to the wild type. Scale bar 1 mm. **g** Representative µCT cross-sections showing osteosclerotic lesions and the ivory region (arrowhead) in the L4 vertebra of a *Zfp687*^P937R/+^ mutant compared to the wild type. Scale bar 1 mm. **h** Representative µCT cross-sections showing chaotic structure and trabecularization (arrowhead) of the femoral cortical bone in a *Zfp687*^P937R/+^ mutant compared to wild type. Scale bar 1 mm. **i** Histological sections of pagetic lesions in *Zfp687*^P937R/+^ femurs, showing woven bone through H&E staining (left) and polarized-light microscopy (right)

Next, we performed skeletal phenotyping of 16-month-old mice, the equivalent of 55-year-old humans, a state of full-blown pathology.^14,20,25^ Remarkably, 87% of aged *Zfp687*^P937R/+^ and *Zfp687*^P937R/P937R^ mice developed polyostotic osteolytic-like lesions, affecting the lumbar spine and the calvarial bones, sites usually affected in pagetic patients (Fig. 6a–c). Three-dimensional reconstruction from µCT analyses also revealed the formation of large protruding osteophytes at the medial and lateral knee joint margins in 8 out of 16 mutant animals (Fig. 6d). These ectopic outgrowths, although less frequent, were also identified at the spine (Fig. 6e). Altogether, osteophytes made the movement of affected mice slow and difficult (Supplementary Movie S1). In fact, bidimensional µCT reconstruction highlighted enlargement of the distal epiphysis of femurs and structural changes in the subchondral trabecular bone microstructure affected by osteophyte formation (Fig. 6f, arrowhead). The occurrence of osteophytes together with vertebral fusion (shown in Fig. 6a, b) is compatible with an ongoing osteoarthritic process.^26^

Consistent with a pagetic phenotype, µCT scanning of 16-month-old mice also revealed osteosclerotic lesions in the lumbar vertebrae (Fig. 6g) and distal epiphyses of femurs (Fig. 6h) of mutant mice. We detected enlarged bones with ivory regions and trabecularization of cortical bone (Fig. 6h, arrowhead). Histological analysis of these lesions showed an increase in bone resorption and formation with accumulation of woven bone, as detected by polarized light microscopy (Fig. 6i). The frequency and type of skeletal defects detected in aged mice are reported in Table 1. Thus, altogether, these results illustrate that the P937R mutation is necessary and sufficient to fully develop a severe form of PDB-like.

**Table 1:** Phenotypic analysis of skeletal alterations in 16-month-old mice

	osteolysis in appendicular skeleton	osteolysis in axial skeleton	osteosclerosis in appendicular skeleton	osteosclerosis in axial skeleton	osteophyte at knee joint	osteophyte at spine	at least 2 sites affected	at least 3 sites affected
Wild type	0%	11%	0%	0%	0%	0%	0%	0%
*Zfp687*^P937R/+^	22%	70%	11%	20%	67%	30%	80%	45%
*Zfp687*^P937R/P937R^	40%	60%	0%	0%	0%	0%	60%	20%

### *Zfp687* is an essential driver of osteoclast differentiation

**Fig. 7:**
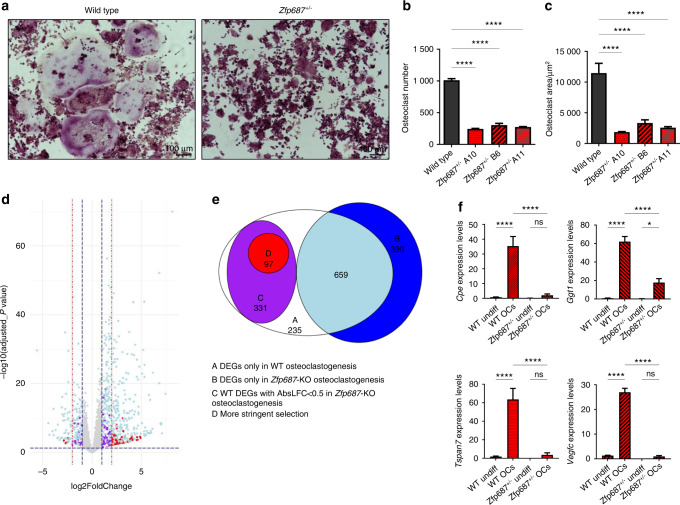
The pivotal role of *ZNF687* in proper osteoclast differentiation. **a** Representative images of TRAP-stained osteoclasts from wild-type and *Zfp687*^*+/−*^ RAW264.7 cells after 5 days of sRANKL stimulation. **b** Bar graph showing the mean number of TRAP^+^ osteoclasts (more than 3 nuclei/cell) in wild-type (*n* = 2) and *Zfp687*^*+/−*^ (*n* = 6) clones. Data are presented as the mean ± s.d. Statistical significance was assessed by one-way ANOVA with Dunnett’s multiple comparison test (****P < 0.000 1). **c** Bar graph showing the mean area of TRAP^+^ osteoclasts in wild-type and *Zfp687*^*+/−*^ clones. **d** Volcano plot showing the distribution of differentially expressed genes (DEGs) between wild-type RAW264.7 and *Zfp687*^*+/−*^ clones. In light blue, DEGs shared by wild type and *Zfp687*-KO osteoclastogenesis; in purple, DEGs in wild type but unchanged in *Zfp687*-KO osteoclastogenesis (log2foldchange ≥ 1 and ≤ −1); in red, selected DEGs in wild type but unchanged in *Zfp687*-KO osteoclastogenesis (log2foldchange ≥ 2 and ≤ −2). **e** Venn diagram of DEGs in wild-type and *Zfp687*-KO osteoclastogenesis. **f** Bar graphs showing the relative expression of genes selected for RNA-seq validation. Undiff: undifferentiated; OCs: osteoclasts. Data are presented as the mean ± s.d. Statistical significance was assessed by one-way ANOVA with a multiple comparison test (*P < 0.05; ****P < 0.000 1)

To obtain mechanistic insights into the role of the *Zfp687* gene in bone metabolism, we induced CRISPR/Cas9-mediated *Zfp687* knockout in the murine RAW264.7 macrophage cell line. We selected three different heterozygous clones (*Zfp687*^*+/−*^). TRAP staining performed after 5 days of sRANKL stimulation revealed that osteoclast formation and differentiation were severely impaired in *Zfp687*^*+/−*^ cells (Fig. 7a). Indeed, the number of mature osteoclasts, identified as TRAP-positive cells with more than 3 nuclei, dramatically decreased by an average of 73% in all KO clones analyzed compared to the wild-type counterparts (Fig. 7b). Moreover, we observed that *Zfp687*^*+/−*^ osteoclasts showed a strongly reduced surface area (average 3 800 µm^2^), while wild-type cells were typically larger (average 11 000 µm^2^) (Fig. 7c). This result indicates that a single copy of the Zfp687 transcription factor is not sufficient to drive proper osteoclastogenesis upon sRANKL stimulation.

To identify Zfp687 target genes that may influence the correct process of osteoclast differentiation, we performed RNA sequencing on RNA extracted from wild-type RAW264.7 cells and three *Zfp687*^*+/−*^ clones before and after RANKL osteoclastogenic induction for 5 days. Under physiological conditions, i.e., in wild-type extracts, a total of 1 322 genes were differentially expressed during the process of osteoclast differentiation: 1 055 upregulated and 267 downregulated genes (Fig. 7d). CLEAR (coordinated lysosomal expression and regulation) signaling, which regulates lysosomal biogenesis and function, was the top upregulated pathway (*P* = 5.24E-10).^27^ Conversely, transcriptomic profiling of the osteoclast differentiation process in *Zfp687*^*+/−*^ cells highlighted that 381 genes previously detected as upregulated in the wild-type context remained unchanged (Fig. 7e, purple). Similarly, 47 genes previously found to be downregulated during control osteoclastogenesis were unchanged in the three mutant processes (Fig. 7d). These data suggest that these genes, whose expression was unperturbed by RANKL stimulation in *Zfp687*^*+/−*^ cells, might be under the transcriptional control of Zfp687. We observed that the crucial genes for osteoclastogenesis, including *Ctsk*, *Acp5* and *Mmp9,* were significantly upregulated in both control and mutated processes, confirming the phenotypic evidence that mutant osteoclastogenesis was severely impaired but not completely abolished. Therefore, we focused on the genes that remained unchanged in *Zfp687*^*+/−*^ cells after stimulation. By using a more stringent cut-off parameter, we restricted our analysis to 92 upregulated and 5 downregulated genes (Fig. 7e, red). From this list, we selected the top differentially expressed genes and those with a clear involvement in osteoclast differentiation, obtaining a high-ranking list of 16 genes (15 up- and 1 downregulated) (Table 2). Among them, *Tspan7*, *Cpe*, *Vegfc*, and *Ggt1* were previously found to have a role in osteoclastic differentiation.^28–34^ We confirmed through real-time PCR that their expression only increased in physiological osteoclastogenesis and remained unchanged in stimulated *Zfp687*^*+/−*^ cells (Fig. 7f). In conclusion, we demonstrated that the Zfp687 transcription factor is a crucial regulator of osteoclast differentiation by regulating several important genes whose study could allow us to discover further molecular mechanisms at the base of the PDB.

**Table 2:** Information of 16 differential expressed osteoclastic genes associated with the loss of *Zfp687* gene

Gene	Description	Gene ID	log2FoldChange in wild type OCs	*P*-value	log2FoldChange in *Zfp687*^*+/−*^ OCs	Function
*TSPAN7*^10,20^	tetraspanin 7	ENSMUSG00000058254	5,804371453	2,04E-06	0,056380196	regulates actin ring formation necessary for the bone resorbing activity of osteoclasts
*MSLN*	mesothelin	ENSMUSG00000063011	4,913852881	6,01E-05	0,192468283	
*DOK7*	docking protein 7	ENSMUSG00000044716	4,573557479	3,37E-05	0,114239406	
*SLC4A3*	solute carrier family 4, member 3	ENSMUSG00000006576	4,280105079	1,33E-04	0	
*AEBP1*	AE binding protein 1	ENSMUSG00000020473	4,181308745	1,73E-04	0,284734917	
*CPE*^21^	carboxypeptidase E	ENSMUSG00000037852	4,141846373	1,49E-04	0,483595016	prohormone-processing enzyme upregulated during osteoclast differentiation induced by RANKL
*VEGFC*^22,23^	vascular endothelial growth factor C	ENSMUSG00000031520	4,027854023	2,38E-05	0,148573253	regulating osteoclast activity through an autocrine mechanism
*GGT1*^24,25^	gamma-glutamyltransferase 1	ENSMUSG00000006345	4,015403244	1,55E-05	0,432081647	induces the osteoclast formation independently of its enzymatic activity, by acting as a local cytokine
*Nrgn*	neurogranin	ENSMUSG00000053310	3,845659324	4,21E-04	0,263986934	
*AQP9*	aquaporin 9	ENSMUSG00000032204	3,257156697	1,25E-03	0,336258443	
*CA6*	carbonic anhydrase 6	ENSMUSG00000028972	3,060020914	1,54E-03	0,194646503	
*CTH*	cystathionine gamma-lyase	ENSMUSG00000028179	2,600514372	3,43E-04	0,330195106	
*ANO7*	anoctamin 7	ENSMUSG00000034107	2,520390607	9,59E-08	0,416112132	
*C22orf23*	chromosome 22 open reading frame 23	ENSMUSG00000033029	2,157503749	9,05E-05	0,473352822	
*NUPR1*	nuclear protein 1, transcriptional regulator	ENSMUSG00000030717	2,00965636	4,95E-05	0,17320238	
*PDCD1*	programmed cell death 1	ENSMUSG00000026285	−2,426926091	8,54E-05	−0,409674667	

### *Zfp687*^P937R^ drives hepatocellular carcinoma in PBD model mice

**Fig. 8:**
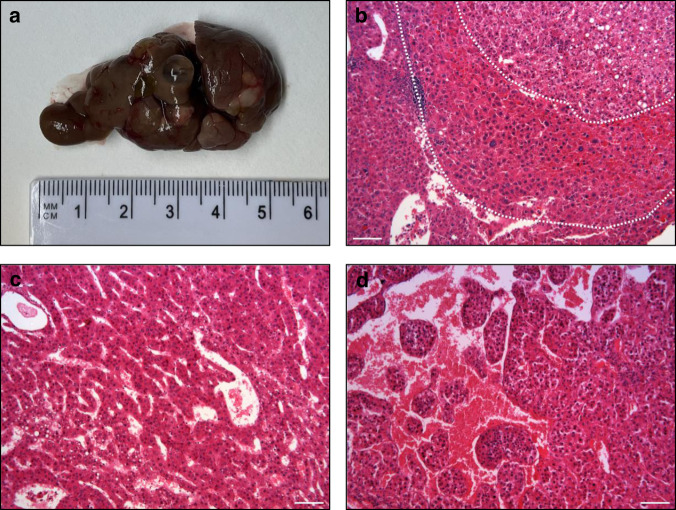
Histological characterization of hepatic nodules. **a** Gross appearance of a mutant *Zfp687* liver. **b** Nodule-in-nodule HCC, (**c**) Trabecular HCC, (**d**) Macrotrabecular HCC with peliosis-like sinusoids. Scale bar, 100 µm

Since PDB patients with the *ZNF687* mutation are prone to developing giant cell tumor degeneration, we looked for bone tumors in *Zfp687* mutant mice. Up to 24 months of age, this model did not develop any bone tumors. However, mutant mice still seem to be predisposed to tumorigenesis because 2 out of 6 heterozygous and 5 out of 11 homozygous mutant mice at 20 months of age developed multiple macroscopic hepatic nodules. We detected an average of 9 nodules/mouse (95% CI: 2–17), with a size of 46.41 ± 36.30 mm^2^ (95% CI: 36.7–56.1) (Fig. 8a). Histological characterization revealed that these nodules were fully developed hepatocellular carcinomas with occasional nodule-in nodule appearance (Fig. 8b), frequently hemorrhagic with large vascular lacunae, and commonly of trabecular or compact histotypes (Fig. 8c, d). Interestingly, the tissue architecture often appears severely deranged, with peliosis-like dilated sinusoids and loss of cell cohesiveness (a feature of more aggressive tumors in the Edmondson-Stainer score), even in the presence of low nuclear atypia and a conserved nuclear/cytoplasmic ratio (suggestive of well to moderately differentiated tumors in the WHO score) (Table 3).

**Table 3:** HCC histological classification according to WHO and Edmondson-Steiner grade systems

WHO scoring	Well	Moderate	Poor	
*Zfp687*^**P937R/+**^	1 (20%)	4 (80%)	0 (0%)	
*Zfp687*^**P937R/P937R**^	7 (25%)	14 (50%)	7 (25%)	
Nodule n. (%)	8 (24%)	18 (55%)	7 (21%)	
ES scoring	G1	G2	G3	G4
*Zfp687*^**P937R/+**^	0 (0%)	0 (0%)	5 (100%)	0 (0%)
*Zfp687*^**P937R/P937R**^	0 (0%)	12 (43%)	9 (32%)	7 (25%)
Nodule n. (%)	0 (0%)	12 (36%)	14 (42%)	7 (21%)

## Discussion

Paget’s disease of bone (PDB) is a focal bone remodeling disorder in which osteoclasts appear giant-sized with increased bone destruction activity, and osteoblasts follow this process through their disorganized bone deposition activity.^2^ As a result, the remodeled bone becomes weaker, deformed and more likely to fracture. Previously, we described that the founder P937R mutation in the *ZNF687* gene causes a severe form of PDB complicated by giant cell tumor degeneration (GCT/PDB).^14,20,35^ To determine the effect of the P937R mutation on bone metabolism and PDB pathogenesis, we generated the *Zfp687* knock-in mouse model. Mice harboring the mutation, in heterozygosity and homozygosity, develop an impressive skeletal phenotype that worsens as they age, mirroring the late onset of PDB in humans. Specifically, µCT analyses performed on adult and aged mutant mice displayed bone remodeling alterations starting from 8 months of age, affecting both the axial and appendicular skeleton. This phenotype was highly pervasive at 16 months of age, in agreement with the age of appearance of full-blown disease in human patients. Of note, unlike PDB patients, adult knock-in mice show generalized trabecular bone loss, reminiscent of an osteoporotic phenotype. Nonetheless, mutant mice exhibit focal osteosclerotic lesions, indicating that focal bone remodeling alterations occur on a background of global bone reduction. Furthermore, adult mutant mice displayed increased osteoid thickness, which was not observed in younger mice and hence could likely be due to an enhanced rate of deposition of bone matrix, which is not yet mineralized, in response to high bone resorption. The ability of matrix mineralization detected ex vivo fully excluded a mineralization defect due to the P937R mutation.

Intriguingly, no P937R homozygous patient has ever been found in our cohort of PDB individuals, leading to the hypothesis that homozygosity for the mutation could be lethal. Nonetheless, homozygous mutant mice are viable and do not show a more aggressive phenotype than heterozygous animals, indicating that the human mutation is so rare that the chance for an individual to inherit two mutated alleles is highly unlikely. This observation also indicates that the inheritance of a single mutated allele is necessary and sufficient to drive the disease.

As PDB is a genetically heterogeneous disease, two other PDB mouse models have been described thus far, harboring the most common *Sqstm1* mutation (P394L) found in human patients.^17–19^ These mutant mice displayed increased osteoclastic resorption,^17^ with typical nuclear inclusion bodies in osteoclasts as well as osteolytic lesions and woven bone.^18,19^ Bone alterations were exclusively detected at the appendicular skeleton, not earlier than 18 months of age. This phenomenon is presumably due to the pleiotropic role of p62, the multifunctional protein encoded by *Sqstm1*, which is involved in multiple cellular functions, such as clearance of misfolded protein, autophagy, cell survival, and regulation of the Keap1–Nrf2 and NFκB pathways.^36^ Another well-established Paget’s disease mouse model is generated by the transgenic expression of the measles virus nucleocapsid (MVNP) in the osteoclast lineage.^37^ MVNP mice developed PDB-like lesions that required MVNP-dependent induction of high IL-6 expression levels in osteoclasts, which in turn resulted in greater expression of IGF-1 and the coupling factor EphrinB2 as well as EphB4 on osteoblasts.^38,39^ Interestingly, this peculiar expression profile was not found in our mouse model, suggesting that various molecular pathways could underlie the occurrence of PDB-like lesions. The *Zfp687* mouse model developed a severe bone phenotype, fully replicating the occurrence of osteolytic, mixed osteolytic/osteosclerotic, and osteosclerotic phases of Paget’s disease. Furthermore, here, we report that *Zfp687* mutants display bone remodeling alterations at the spine, enabling researchers to model and study the disease. This finding is also in agreement with a more severe human PDB when caused by *ZNF687* mutations. In these mice, the disease is so severe that additional complications occur. We indeed found osteophytes at the knee and vertebral joints and vertebral fusion in 50% of aged mutant mice as a consequence of degeneration in osteoarthritis (OA). In fact, OA is a quite common complication of PDB, which becomes even more frequent in *ZNF687*-mutated patients (>40% of cases).^3,14^ In our mouse model, we also highlighted an unexpected increase in BMAT in both heterozygous and homozygous knock-in mice. However, increased marrow fat is not a prominent feature in ordinary human PDB. Therefore, we cannot exclude that this trait could be a peculiarity of *ZNF687*-dependent PDB. Such fat replacement of the bone was also observed in patients with familial expansile osteolysis, a focal bone remodeling disorder with a second peak of onset in elderly individuals, suggesting that BMAT alterations can be observed in bone dysplasia.^40^ In contrast, two different studies independently reported that BMAT-derived RANKL induces osteoclastogenesis and bone remodeling, indicating that excessive RANKL generated by bone marrow adipocytes is an underlying cause of skeletal disorders.^41,42^ Therefore, we cannot exclude a positive effect of the mutation on BMAT that further enhances osteoclast differentiation and therefore results in a more aggressive phenotype. Our data also indicated that BM-MSCs harboring the P937R mutation are capable of differentiating toward both osteoblast and adipocyte cell lineages with a higher efficiency than control cells. To explain this apparently counterintuitive phenomenon, which counteracts the mutually exclusive differentiation program described for fate-decision in one of the two different lineages,^43^ we could speculate that bone marrow of mutated mice might be enriched either in progenitors with both osteo- and adipo-differentiation properties or in MSCs with an increased proliferative ability. Thus, additional studies are needed to better understand the involvement of Zfp687 during BM-MSC fate commitment and differentiation.

In this study, we also reported a set of genes involved in osteoclastogenesis under the control of Zfp687. Among them, *Tspan7*, *Cpe*, *Vegfc*, and *Ggt1* are described as having a role in regulating the bone-resorbing function of osteoclasts.^28–34^ Of course, additional functional studies are necessary to determine the specific effect of these genes on PDB pathogenesis or to identify additional molecular mechanisms. Finally, an open question related to this model remains the development of the tumor. PDB patients with the *ZNF687* mutation, if untreated, undergo giant cell tumor degeneration. Up to 24 months of age, this model did not develop any bone tumors. Nonetheless, the evidence that mutants of Zfp687 did not drive GCT tumorigenesis within 24 months of age is not surprising: a single genetic alteration is frequently not sufficient to lead to a malignant phenotype in mice or otherwise with a lower penetrance than typically seen in humans.^44^ This phenomenon could be related to the notion that cancer is a multistep process, and more genetic and environmental events may be necessary for its development. However, mutant mice still seem to be predisposed to tumorigenesis because mutant mice at 20 months of age exhibited fully developed hepatocellular carcinomas (HCC). This result was consistent with the role for ZNF687 as an oncogene found in HCC.^45^ It remains to be investigated whether the development of HCC is due to biochemical alterations similar to those underlying the bone phenotype.

In conclusion, our *Zfp687* mouse model provides a new tool to study and treat Paget’s disease of bone and its related complications, and functional analyses derived from RNA-seq data will enable a deep understanding of the transcriptional network regulated by Zfp687.

## Materials and methods

### Generation of the *Zfp687*^P937R^ knock-in mouse model

To generate the PDB mouse model carrying the P937R mutation in the *Zfp687* gene, we adopted the homologous recombination strategy (Fig. S1a). Human and mouse gene homology was verified through the mVista bioinformatics tool (https://genome.lbl.gov/vista/index.shtml). A BAC library was used as a template to amplify the *Zfp687* locus for homologous recombination. PCR fragments were cloned into the pGND vector as long and short homology arms. The P937R mutation (c.2810C>G) was introduced in the long homology arm by PCR-mediated mutagenesis (QuikChange Lightning, Agilent) using the following primers: sense 5′-GTTGGTCGGGGTCGCTCAGGGGAGC-3′; antisense 5′-GCTCCCCTGAGCGACCCCGACCAAC-3′. Targeting of the construct was performed in the E14Tg2a embryonal stem (ES) cell line, and targeted ES cell clones were identified by Southern blot analysis using a 3′ probe and an internal Neo probe on SphI-cut genomic DNA and a 5′ probe on BglII-cut genomic DNA. Sanger sequencing confirmed the presence of the c.2810C>G mutation. One *Zfp687*-mutated ES clone was injected into C57BL6 blastocysts to establish a mutant mouse colony. The flox-flanked neomycin resistance gene was removed by crossing the mice with a Cre transgenic mouse. These mice were generated on a B6D2F1/J background and maintained on a mixed genetic background of C57BL/6J and DBA/2J. Genotyping was performed with allele-specific primers: forward 5′-GACAGCCCTCTAAACCTCAAGACC-3′; reverse 5′-AGCAGGAGCATTAGTGTTGGATTC-3′, leading to the amplification of two different sized products depending on the presence or absence of the loxP site. Animals were handled in accordance with authorization no. 125-2021-PR released by the Italian Ministry of Health; all mice were housed in a pathogen-free barrier environment.

### Microcomputed tomography (μCT) analysis

Male mice were sacrificed by CO_2_ inhalation at the indicated age. The skin was removed, and femurs, tibiae, spine, and skull were cleaned from adherent and soft tissue, fixed in 4% paraformaldehyde, PFA (Sigma‒Aldrich, #158127) for 24 h at 4 °C, and then stored in 70% ethanol. μCT analyses were conducted using the SCANCO Medical-μCT40 (Scanco Medical AG, Bassersdorf, Switzerland). For bone morphometry, femoral trabecular and cortical bone were scanned using the following parameters: E = 70 kV; I = 114 μA; integration time of 600 ms; 3 μm isotropic voxel size. Femurs were scanned 1 mm from the distal board of the growth plate. Cortical thickness was measured at the midshaft region of the femur diaphysis. For femur trabecular bone and for midshaft cortical bone, 209 and 36 slices were analyzed, respectively. Vertebral trabecular bone was analyzed by selecting and scanning the whole L4 vertebra, using the last rib-bearing thoracic vertebra as a reference, with the following parameters: E = 55 kV; I = 145 μA; integration time of 300 ms; and 12 μm isotropic voxel size. The trabecular bone of the vertebral body was evaluated immediately below the superior growth plate. A lower threshold of 270 was used for the evaluation of all scans. For trabecular bone of the femur and L4 vertebra, the structural parameters bone volume/total volume (BV/TV), trabecular thickness (Tb.Th), trabecular number (Tb.N), and trabecular separation (Tb.Sp) were considered. For reconstruction of solid 3D images, selected bone samples were scanned at high resolution. Femur, lumbar spine, and skull samples were scanned using the following parameters: E = 70 kV; I = 114 μA; integration time of 300 ms; 6 μm isotropic voxel size. The reconstructed solid 3D images were applied to visualize bone morphology and microarchitecture.

### Histological analysis

For histology, long bones (femurs and tibiae) and the lumbar region of the spine were decalcified in 14% ethylenediaminetetraacetic acid (EDTA, Sigma–Aldrich, #27285) for 14 days, replacing the solution every 3 days. Then, bone samples were dehydrated with an ethanol series (70%, 80%, 90%, 100% Et-OH), treated with xylene, and paraffin-embedded. Bone slices of 3 and 5 μm were obtained by manual microtome. Bone sections were subjected to a wax-removal procedure by xylene treatment and rehydration through a graded series of alcohol (100%, 90%, 80%, 70% Et-OH) and tap water. Bone sections were stained with hematoxylin and eosin (H&E) for general tissue morphology and with tartrate-resistant acid phosphatase (TRAP) (Sigma‒Aldrich, #387A) to detect osteoclast activity, according to standard protocols. After staining, bone sections were dehydrated, mounted with mounting medium (Bio-Optica, #05-BMHM), covered with a coverslip, and analyzed by transmission light microscopy using a Nikon Motorized Eclipse Ni-U Microscope and Nikon Manual Optical Microscope. Osteoclast surface and number were measured by TRAPHisto open-source software.^46^ For osteoblast quantification (Ob.N/BS), paraffin-embedded tibial samples were stained with H&E. Osteoblasts were identified as cuboidal cells lining the trabecular bone surface in the proximal metaphyseal region of tibiae. Osteoblasts were manually counted from 5 fields, 2 slices per animal (20X magnification), and the mean was calculated for each animal (*n* = 5 wild type; *n* = 4 *Zfp687*^P937R/+^; *n* = 7 *Zfp687*^P937R/P937R^). The bone surface was determined by ImageJ software. For BMAT quantification and adipocyte measurements, H&E staining was performed on tibiae. Adipocyte cells were identified by their thin cytoplasmic layer that lines the lipid droplet and forms the ghost-like remnant of the adipocytes. Adipocyte ghost cells were manually counted from at least 3 sections spaced 100 µm apart per animal (4X magnification) (*n* = 7 wild type; *n* = 6 *Zfp687*^P937R/+^; *n* = 8 *Zfp687*^P937R/P937R^). All adipocytes in each section were counted, and 160 adipocytes/section were measured for area evaluation. All measurements were determined using ImageJ software.

Liver tissues were collected and fixed in 4% PFA for 24 h at 4 °C and then stored in 70% ethanol. Then, samples were dehydrated with an ethanol series (70%, 80%, 90%, 100% Et-OH), treated with xylene, and paraffin-embedded. Hepatic sections of 5 μm were obtained by manual microtome and were subjected to wax-removal procedure by xylene treatment and rehydration through a graded series of alcohol (100%, 90%, 80%, 70% Et-OH) and tap water. Liver histology was assessed on H&E-stained sections. Tumor nodules were characterized using the WHO (“Classification of Tumours of the Digestive System”^47^ and Edmondson and Steiner^48^ grading systems.

### Von Kossa/van Gieson staining and osteoid quantification

For osteoid and matrix mineralization evaluation, 7 μm sections from methyl methacrylate (MMA)-embedded femurs of 3- and 8-month-old mice were stained with von Kossa and counterstained with van Gieson, according to standard procedures. Briefly, aqueous silver nitrate solution was added to the slides, which were then incubated with soda-formol solution for 5 min. Then, sodium thiosulfate was added and incubated for 5 min to remove unreacted silver. Von Kossa-stained samples were rinsed in tap water and counterstained with van Gieson solution for 30 min. Slices were mounted and covered with a coverslip and analyzed by transmission light microscopy. Osteoid quantification was performed using the threshold color function of ImageJ software.

### Generation of *Zfp687* knockout RAW264.7 cell clones using CRISPR‒Cas9 technology

*Zfp687* knockout RAW264.7 cell clones were obtained by CRISPR‒Cas9 technology. The small guide RNA (sgRNA) was designed using the tool at http://crispor.tefor.net/, targeting exon 2 at the fourth ATG, predicted to be surrounded by a Kozak consensus sequence (sense 5′-CACCGCCTCAAGGGGCCTTGAAAC-3′). The sgRNA was cloned into the pSpCas9(BB)-2A-GFP plasmid (Addgene, #48138). Then, the genetic transformation of RAW264.7 cells was obtained by nucleofection using the Amaxa Cell Line Nucleofector Kit V (Lonza) for RAW264.7 and following the protocol for Amaxa Nucleofector. After 24 h, single GFP-positive cells were sorted in 96-well plates with Becton Dickinson FACSAria III system. We obtained three heterozygous *Zfp687* knockout clones; homozygous knockout clones were never detected. Clones harboring distinct heterozygous frameshift mutations (*Zfp687*^*+/−*^) were confirmed by Sanger sequencing.

### Cell culture

Primary murine bone marrow-derived mesenchymal stromal cells (BM-MSCs) were obtained from femurs and tibiae of 8-week-old mice, adapted from the method described in.^49^ Briefly, mice were euthanized through CO_2,_ and immediately after sacrifice, femurs and tibiae were carefully cleaned of all connective tissues; both the distal and proximal ends of bones were cut, and the marrow was centrifuged out. ACK (ammonium-chloride-potassium) lysing buffer was used to eliminate red blood cells. Total bone marrow cells were cultured in complete expansion medium (MesenCult Expansion Kit (Mouse) #05513, Stem Cell Technologies) at 37 °C and 5% CO_2_. BM-MSCs were expanded for 7 days. For osteogenic differentiation, cells were detached using 0.25% trypsin-EDTA, plated in 24-well plates and cultured in complete expansion medium until they reached 80%–90% confluency. Then, for osteogenic differentiation, the medium was replaced with complete MesenCult Osteogenic Medium (MesenCult Osteogenic Stimulatory Kit (Mouse) #05504, Stem Cell Technologies), and the cells were cultured at 37 °C and 5% CO_2_. The medium was changed every 3 days for 8 days. Differentiated osteoblasts were fixed in 70% ethanol and stained with Alizarin Red Solution (Sigma‒Aldrich #A5533). Destaining was conducted to quantitatively determine mineralization by adding acetic acid. Absorbance was measured in the microplate reader PerkinElmer luminometer (Victor X3) at 405 nm.

For adipogenic differentiation, the medium was replaced with MesenCult Adipogenic Differentiation Medium (Mouse) #05507 (Stem Cell Technologies) for 6 days. Adipogenic differentiation was assessed by Oil Red O (ORO) staining (Sigma‒Aldrich #O1392) following the manufacturer’s instructions. For quantification, ORO was extracted by adding isopropanol, and absorbance was read in the microplate reader PerkinElmer luminometer (Victor X3) at 490 nm.

RAW264.7 cells were cultured in DMEM High Glucose GlutaMAX (Gibco) with 10% FBS, 1% penicillin/streptomycin, and 1% L-glutamine at 37 °C and 5% CO_2_. For osteoclast differentiation, 5 × 10^3^ cells were plated in 24-well plates, and the medium was switched to Minimum Essential Medium α (MEM- α) GlutaMAX (Gibco) with 10% FBS, 1% penicillin/streptomycin, and 1% L-glutamine. The next day, the medium was changed and supplemented with 100 ng·mL^−1^ sRANKL (Peprotech) for osteoclastogenic induction. The medium was changed every 48 h until the end of the differentiation (5 days upon stimulation). Differentiated osteoclasts were fixed in 4% PFA and stained with tartrate-resistant acid phosphatase (TRAP) (Sigma‒Aldrich).

### Protein extraction and Western blotting

Total protein extraction from murine cell lines (RAW264.7 cells and osteoclast-derived cells) was performed in RIPA buffer (50 mmol·L^−1^ Tris–HCl pH 7.5; 150 mmol·L^−1^ NaCl; 1 mmol·L^−1^ DTT; 50 mmol·L^−1^ sodium fluoride; 0.5% sodium deoxycholate; 0.1% SDS; 1% NP-40; 0.1 mmol·L^−1^ phenylmethanesulfonylfluoride; 0.1 mmol·L^−1^ sodium vanadate) with 1X proteinase inhibitor cocktail (Applied Biological Materials #G135). Protein quantification was obtained by the Bradford method (Bio-Rad #5000006). Protein samples were boiled at 95 °C for 5′ and then separated by SDS‒PAGE electrophoresis using 8%–16% Tris-glycine gels (Invitrogen #XP08160). Samples were transferred to a nitrocellulose membrane (Invitrogen #IB23002) and blocked with 4% w/v nonfat dry milk dissolved in TBS-T (1X TBS, 0.05% Tween-20) for 1 h at RT. Primary antibodies used for the Western blot experiments were rabbit anti-ZNF687 (1:3 000, Novus NBP2-41175), mouse anti-β-actin (1:10 000, Santa Cruz #47778), and mouse anti-α-tubulin (1:15 000, Sigma‒Aldrich #T6074). Membranes were incubated with secondary antibodies conjugated with HRP for 1 h at RT. The bands were visualized using enhanced chemiluminescence detection reagents (Advansta #K-12043-D10) and autoradiographic films (Aurogene #AU1101). Equal loading was confirmed by using antibodies against β-actin and α-tubulin. The intensity of the Western blot signals was determined by densitometric analysis using ImageJ software and normalized to the density value of the loading control. Protein extracts of peripheral blood mononuclear cells (PBMCs) and differentiated osteoclasts from a healthy donor and P937R-mutated patient were previously collected^20^ and already present in our laboratory.

### RNA isolation and qRT‒PCR analysis

Total RNA extraction (from BM-MSCs, mouse osteoblasts, RAW264.7 cells, and differentiated osteoclasts) was obtained using TRI Reagent (Sigma‒Aldrich #T9424) following the manufacturer’s instructions. One microgram of total RNA was reverse transcribed into cDNA using the RevertAID RT kit (Thermo Fisher #K1622). qRT‒PCR was performed using SYBR Select Master Mix for CFX (Applied Biosystems) and specific primers (listed in Table 4) on a CFX Opus RT PCR System instrument. The transcript levels were normalized to the levels of *Gapdh* within each sample, and the ΔΔCT method was used. The reaction was conducted in triplicate. cDNA of PBMCs and differentiated osteoclasts derived from healthy donors and P937R-mutated patients was already present in our laboratory.

**Table 4:** Sequence of primers used for qRT-PCR

Gene	Sequence 5′-3′
*CTSK*	F: GTCAAAAATCAGGGTCAGTG
	R: GAAGGCATTGGTCATGTAGC
*MMP9*	F: CCCGGACCAAGGATACAGTT
	R: TTCAGGGCGAGGACCATAGA
*TRAP*	F: TTCTCTGACCGCTCCCTTCG
	R: AGGCTGCTGGCTGAGGAAGT
*ZNF687*	F: AGGCCAAGCTGATCTACAAG
	R: GATGGGTGTTCTTGAGATGT
*Cpe*	F: CGAGCTGAAGGACTGGTTTG
	R: GGGGCAAGCTTTGAATTTTGG
*Ggt1*	F: CTCAGAGATTGGACGGGATA
	R: GTGCTGTTGTAGATGGTGAAG
*Tspan7*	F: CTGTCTCAAAACCCTCCTCA
	R: GAGGCCAAAAACCACGATG
*Vegfc*	F: GATGTGGGGAAGGAGTTTGG
	R: TGATTGTGACTGGTTTGGGG
*Zfp687*	F: AAGGAACATGGTAAGTCAGT
	R: GACCTGAACATGCTTCTCCA

### Library preparation and RNA sequencing

The libraries were generated using depleted RNA obtained from 1 μg of total RNA by a TruSeq Sample Preparation RNA Kit (Illumina, Inc., San Diego, CA, USA) according to the manufacturer’s protocol without further modifications. All libraries were sequenced on the Illumina HiSeq 1000, generating 100 bp paired-end reads. Illumina BCL2FASTQ v2.20 software was used for demultiplexing and production of FASTQ sequence files. FASTQ raw sequence files were subsequently quality checked with FASTQC software (http://www.bioinformatics.bbsrc.ac.uk/projects/fastqc). Subsequently, sequences with low quality scores or including adaptor dimers or mitochondrial or ribosomal sequences were discarded from the analysis. The resulting set of selected reads was aligned onto the complete mouse genome using Spliced Transcripts Alignment to a Reference algorithm STAR version 2.7.3 using GRCm39 Genome Assembly and GRCm39.105.gtf as gene definition.^50^ The resulting mapped reads were used as input for the feature Counts function of Rsubread packages and used as gene counts for differential expression analysis using the Deseq2 package.^51^ We used the shrinkage estimator from the apeglm package for visualization and ranking.^52^ Differentially expressed genes (DEGs) were selected based on an adjusted *P* value < 0.05 and by setting Log2FoldChange ≥ 1 for upregulated and ≤ −1 for downregulated genes. The cutoff was further increased to Log2FoldChange ≥ 2 for upregulated and ≤ −2 for downregulated genes for a more stringent filter. Selected DEGs were used as input to perform pathway enrichment analysis by the IPA system (Ingenuity® Systems http://www.ingenuity.com). IPA annotation was used as a starting point for focusing on osteoclast differentiation.

### Quantification and statistical analyses

All data are presented as the mean or median ± standard deviation (s.d.). The sample size for each experiment and the replicate number of experiments are included in the figure legends. Statistical significance was defined as *P* < 0.05. Statistical analyses were performed using ordinary one-way ANOVA or Student’s *t* test (GraphPad Prism; version 9.3.1).

## Supplementary information

Figure S1

Figure S2

Figure S3

Figure S4

Video S1

Supplementary figure and video legends

## Data Availability

All relevant data supporting the key findings of this study are available within the article. Raw sequencing data are available upon request.

## References

[1] Cundy T (2018). Paget’s disease of bone. Metabolism..

[2] Ralston SH (2013). Paget’s disease of bone. N. Engl. J. Med..

[3] Ralston SH (2019). Diagnosis and management of Paget’s disease of bone in adults: a clinical guideline. J. Bone Miner. Res..

[4] Makaram N, Woods L, Beattie N, Roberts SB, Macpherson GJ (2020). Long-term outcomes following total hip and total knee arthroplasty in patients with Paget’s disease of bone (PDB) - a national study. Surgeon.

[5] Roelofs AJ (2020). Identification of the skeletal progenitor cells forming osteophytes in osteoarthritis. Ann. Rheum. Dis..

[6] van der Kraan PM, van den Berg WB (2007). Osteophytes: relevance and biology. Osteoarthr. Cartil..

[7] Altman RD, Collins B (1980). Musculoskeletal manifestations of Paget’s disease of bone. Arthritis Rheum.

[8] Russo S, Scotto di Carlo F, Gianfrancesco F (2022). The osteoclast traces the route to bone tumors and metastases. Front. Cell Dev. Biol..

[9] Deyrup AT (2007). Sarcomas arising in Paget disease of bone: a clinicopathologic analysis of 70 cases. Arch. Pathol. Lab. Med..

[10] Gianfrancesco F (2013). Giant cell tumor occurring in familial Paget’s disease of bone: report of clinical characteristics and linkage analysis of a large pedigree. J. Bone Miner. Res..

[11] Scotto di Carlo F, Whyte MP, Gianfrancesco F (2020). The two faces of giant cell tumor of bone. Cancer Lett..

[12] Hocking LJ (2002). Domain-specific mutations in sequestosome 1 (SQSTM1) cause familial and sporadic Paget’s disease. Hum. Mol. Genet..

[13] Laurin N, Brown JP, Morissette J, Raymond V (2002). Recurrent mutation of the gene encoding sequestosome 1 (SQSTM1/p62) in paget disease of bone. Am. J. Hum. Genet..

[14] Divisato G (2016). ZNF687 mutations in severe paget disease of bone associated with giant cell tumor. Am. J. Hum. Genet..

[15] Scotto di Carlo F, Pazzaglia L, Esposito T, Gianfrancesco F (2020). The loss of profilin 1 causes early onset Paget’s disease of bone. J. Bone Miner. Res..

[16] Rea SL, Walsh JP, Layfield R, Ratajczak T, Xu Jiake J (2013). New insights into the role of sequestosome 1/p62 mutant proteins in the pathogenesis of paget’s disease of bone. Endocr. Rev..

[17] Hiruma Y (2008). A SQSTM1/p62 mutation linked to Paget’s disease increases the osteoclastogenic potential of the bone microenvironment. Hum. Mol. Genet..

[18] Daroszewska A (2011). A point mutation in the ubiquitin-associated domain of SQSMT1 is sufficient to cause a Paget’s disease-like disorder in mice. Hum. Mol. Genet.

[19] Daroszewska A (2018). Zoledronic acid prevents pagetic-like lesions and accelerated bone loss in the p62 P394L mouse model of Paget’s disease. DMM Dis. Model. Mech..

[20] Divisato G, Scotto di Carlo F, Petrillo N, Esposito T, Gianfrancesco F (2018). ZNF687 mutations are frequently found in pagetic patients from South Italy: implication in the pathogenesis of Paget’s disease of bone. Clin. Genet..

[21] Ambrosi TH (2017). Adipocyte accumulation in the bone marrow during obesity and aging impairs stem cell-based hematopoietic and bone regeneration. Cell Stem Cell.

[22] Tencerova M (2018). High-fat diet-induced obesity promotes expansion of bone marrow adipose tissue and impairs skeletal stem cell functions in mice. J. Bone Miner. Res..

[23] Woods GN (2020). Greater bone marrow adiposity predicts bone loss in older women. J. Bone Miner. Res..

[24] Fan Y (2017). Parathyroid hormone directs bone marrow mesenchymal cell fate. Cell Metab..

[25] Dutta S, Sengupta P (2016). Men and mice: Relating their ages. Life Sci..

[26] Fang H, Beier F (2014). Mouse models of osteoarthritis: modelling risk factors and assessing outcomes. Nat. Rev. Rheumatol..

[27] Palmieri M (2011). Characterization of the CLEAR network reveals an integrated control of cellular clearance pathways. Hum. Mol. Genet..

[28] Kwon JO (2016). Tetraspanin 7 regulates sealing zone formation and the bone-resorbing activity of osteoclasts. Biochem. Biophys. Res. Commun..

[29] Kim M (2022). Tetraspanin 7 regulates osteoclast function through association with the RANK/αvβ3 integrin complex. J. Cell. Physiol..

[30] Kim HJ, Hong JM, Yoon HJ, Yoon YR, Kim SY (2014). Carboxypeptidase E is a novel modulator of RANKL-induced osteoclast differentiation. Mol. Cells.

[31] Zhang Q (2008). VEGF-C, a lymphatic growth factor, is a RANKL target gene in osteoclasts that enhances osteoclastic bone resorption through an autocrine mechanism. J. Biol. Chem..

[32] Hominick D (2018). VEGF-C promotes the development of lymphatics in bone and bone loss. Elife.

[33] Niida S (2004). γ-Glutamyltranspeptidase stimulates receptor activator of nuclear factor-κB ligand expression independent of its enzymatic activity and serves as a pathological bone-resorbing factor. J. Biol. Chem..

[34] Hiramatsu K (2007). Overexpression of gamma-glutamyltransferase in transgenic mice accelerates bone resorption and causes osteoporosis. Endocrinology.

[35] Scotto di Carlo, F. et al. ZNF687 mutations in an extended cohort of neoplastic transformations in Paget’s disease of bone: implication for clinical pathology. *J. Bone Miner. Res.* **35**, 1974–1980 (2020). 10.1002/jbmr.3993 32106343

[36] Sánchez-Martın P, Komatsu M (2018). p62/SQSTM1 - steering the cell through health and disease. J. Cell Sci..

[37] Kurihara N (2006). Expression of measles virus nucleocapsid protein in osteoclasts induces Paget’s disease-like bone lesions in mice. J. Bone Min. Res..

[38] Kurihara N (2011). Contributions of the measles virus nucleocapsid gene and the SQSTM1/p62P392L mutation to paget’s disease. Cell Metab..

[39] Teramachi J (2016). Measles virus nucleocapsid protein increases osteoblast differentiation in Paget’s disease. J. Clin. Invest..

[40] Wallace RGH, Barr RJ, Osterberg PH, Mollan RAB (1989). Familial expansile osteolysis. Clin. Orthop. Relat. Res..

[41] Yu W (2021). Bone marrow adipogenic lineage precursors promote osteoclastogenesis in bone remodeling and pathologic bone loss. J. Clin. Invest..

[42] Hu Y (2021). RANKL from bone marrow adipose lineage cells promotes osteoclast formation and bone loss. EMBO Rep..

[43] Bianco P (2013). The meaning, the sense and the significance: translating the science of mesenchymal stem cells into medicine. Nat. Med..

[44] Lehman HL, Stairs DB (2015). Single and multiple gene manipulations in mouse models of human cancer. Cancer Growth Metastasis.

[45] Zhang T (2017). Overexpression of zinc finger protein 687 enhances tumorigenic capability and promotes recurrence of hepatocellular carcinoma. Oncogenesis.

[46] van’t Hof RJ, Rose L, Bassonga E, Daroszewska A (2017). Open source software for semi-automated histomorphometry of bone resorption and formation parameters. Bone.

[47] Nagtegaal ID (2020). The 2019 WHO classification of tumours of the digestive system. Histopathology.

[48] Edmondson, H. A. & Steiner, P. E. Primary carcinoma of the liver a study of 100 cases among 48,900 Necropsies. *Cancer* **7**, 462–503 (1954). 10.1002/1097-0142(195405)7:3<462::aid-cncr2820070308>3.0.co;2-e 13160935

[49] Maridas DE, Rendina-Ruedy E, Le PT, Rosen CJ (2018). Isolation, culture, and differentiation of bone marrow stromal cells and osteoclast progenitors from mice. J. Vis. Exp..

[50] Dobin A, Gingeras TR (2015). Mapping RNA-seq reads with STAR. Curr. Protoc. Bioinforma..

[51] Love MI, Huber W, Anders S (2014). Moderated estimation of fold change and dispersion for RNA-seq data with DESeq2. Genome Biol..

[52] Zhu A, Ibrahim JG, Love MI (2019). Heavy-tailed prior distributions for sequence count data: removing the noise and preserving large differences. Bioinformatics.

