# Supplementary material for: A mutation in the ZNF687 gene that is responsible for the severe form of Paget’s disease of bone causes severely altered bone remodeling and promotes hepatocellular carcinoma onset in a knock-in mouse model
Source: Bone Res. 2023 Mar 14;11:16. doi: 10.1038/s41413-023-00250-3 (PMC10014847; doi:10.1038/s41413-023-00250-3)
Supplement: Supplementary file 1 — Figure S1 [file 41413_2023_250_MOESM1_ESM.pdf]

Figure S1

a

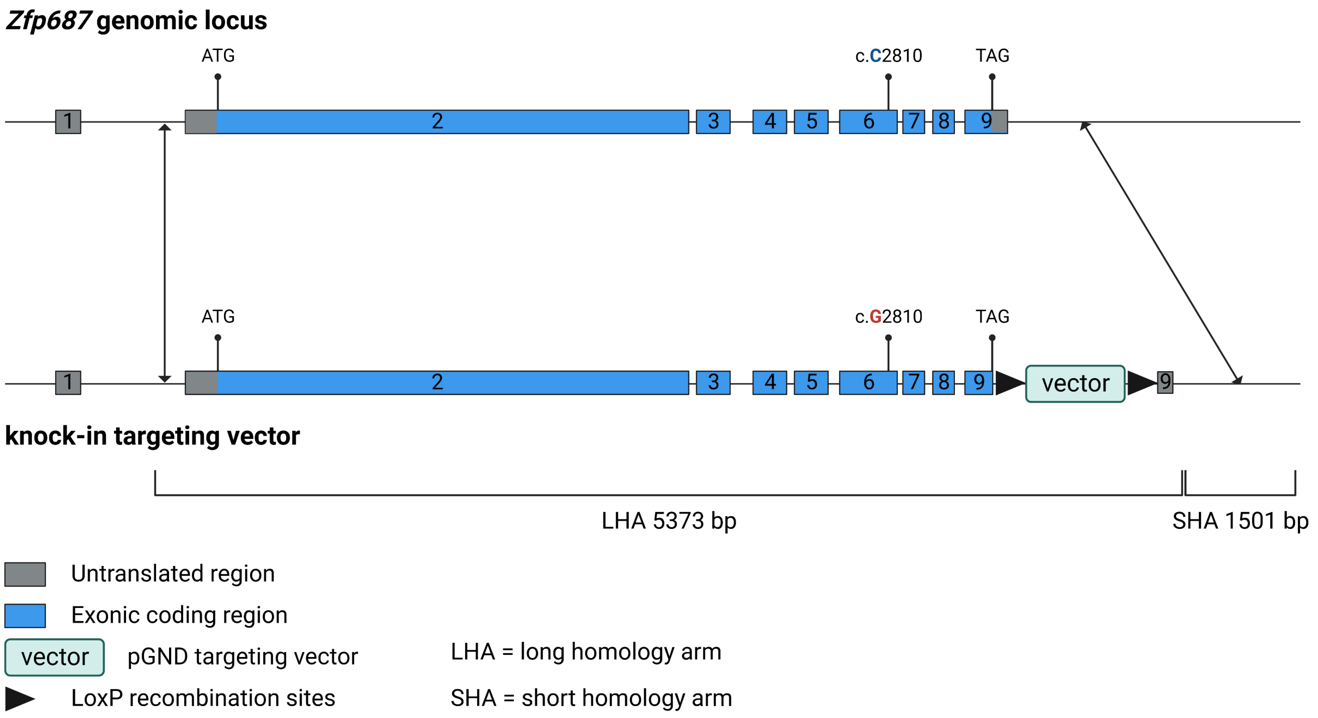
Zfp687 genomic locus
ATG
c.C2810
TAG
1
2
3
4
5
6
7
8
9
ATG
c.G2810
TAG
vector
knock-in targeting vector
LHA 5373 bp
SHA 1501 bp
Untranslated region
Exonic coding region
vector pGND targeting vector
LoxP recombination sites
LHA = long homology arm
SHA = short homology arm

b

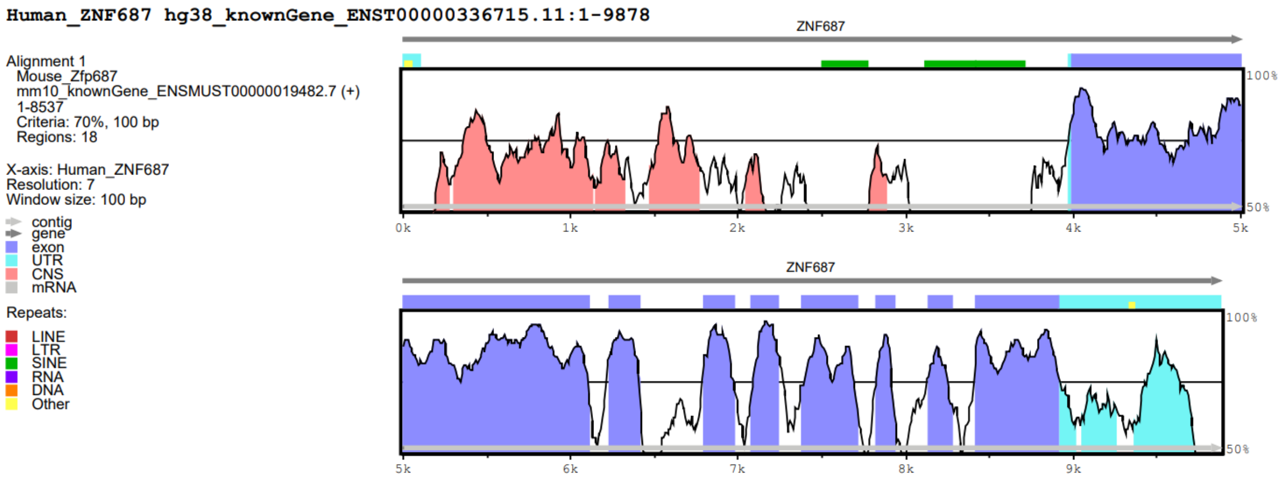
Human_ZNF687 hg38_knownGene_ENST00000336715.11:1-9878
ZNF687
Alignment 1
Mouse_Zfp687
mm10_knownGene_ENSMUST00000019482.7 (+)
1-8537
Criteria: 70%, 100 bp
Regions: 18
X-axis: Human_ZNF687
Resolution: 7
Window size: 100 bp
contig
gene
exon
UTR
CNS
mRNA
Repeats:
LINE
LTR
SINE
RNA
DNA
Other
100%
50%
0k
1k
2k
3k
4k
5k
ZNF687
5k
6k
7k
8k
9k
