# Supplementary figures and images for: A mutation in the ZNF687 gene that is responsible for the severe form of Paget’s disease of bone causes severely altered bone remodeling and promotes hepatocellular carcinoma onset in a knock-in mouse model
Source: Bone Res. 2023 Mar 14;11:16. doi: 10.1038/s41413-023-00250-3 (PMC10014847; doi:10.1038/s41413-023-00250-3)

Figure S2

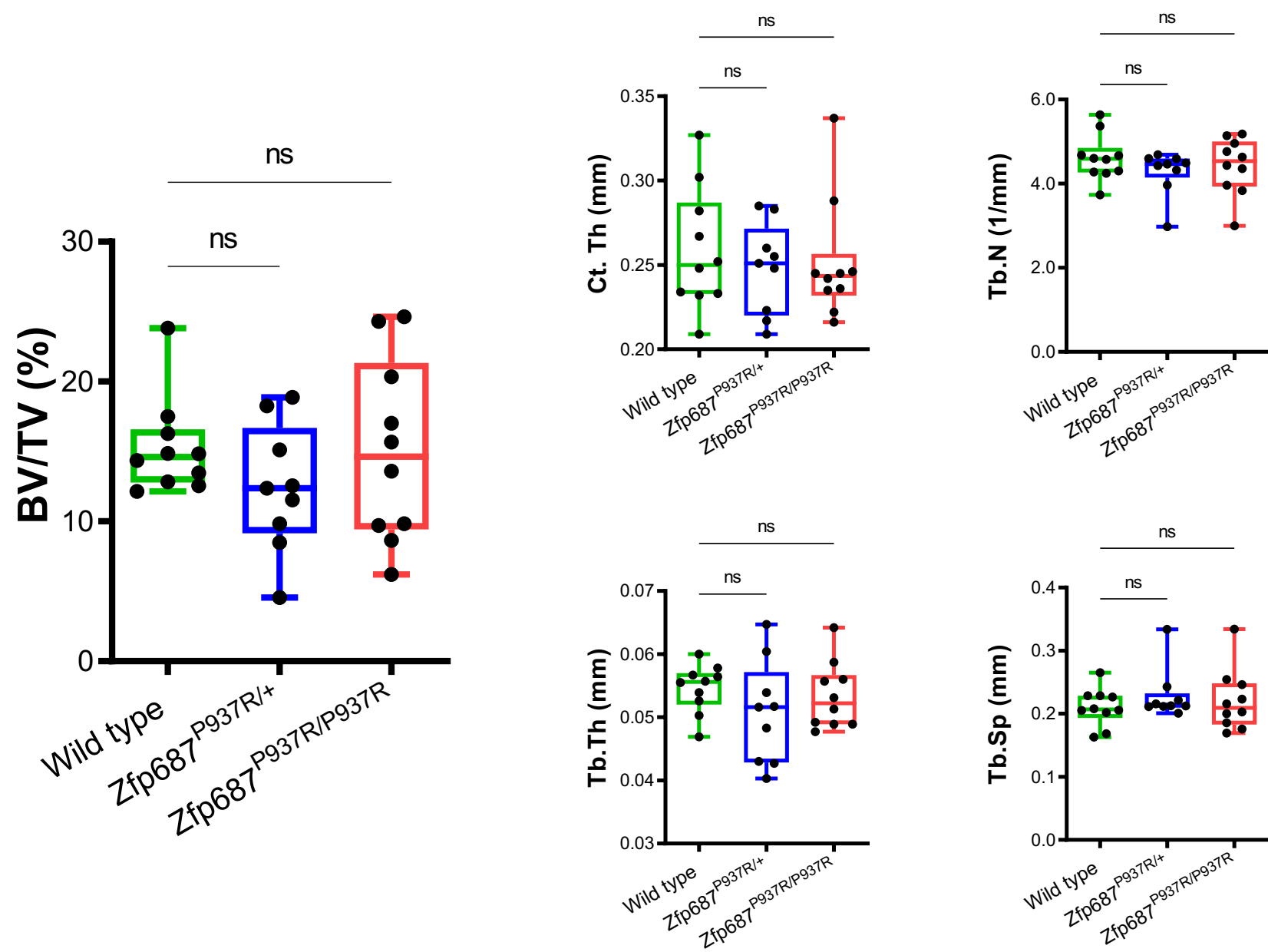

Supplement: Supplementary file 2 — Figure S2 [file 41413_2023_250_MOESM2_ESM.pdf]

Figure S3

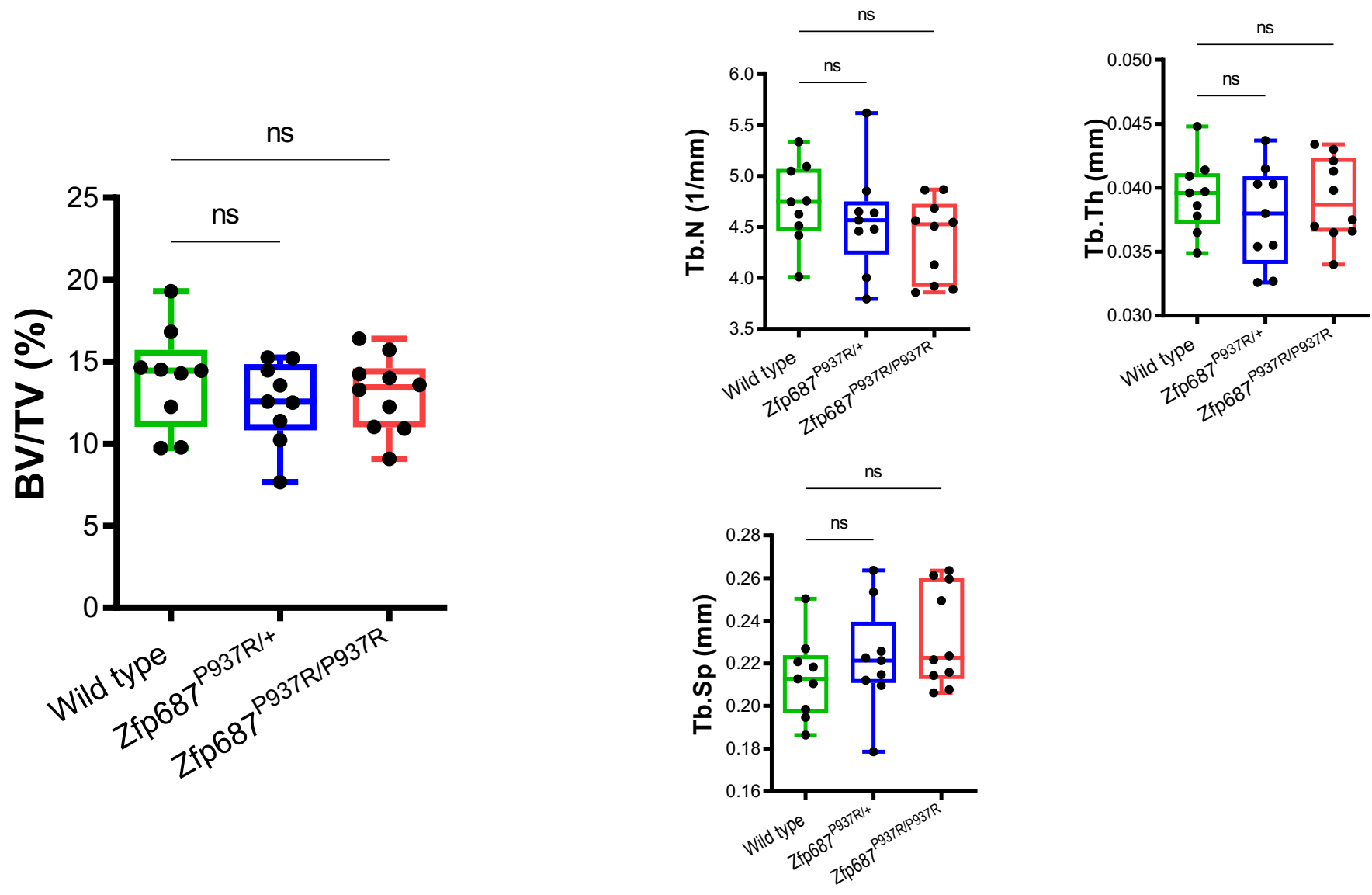

Supplement: Supplementary file 3 — Figure S3 [file 41413_2023_250_MOESM3_ESM.pdf]

Figure S4

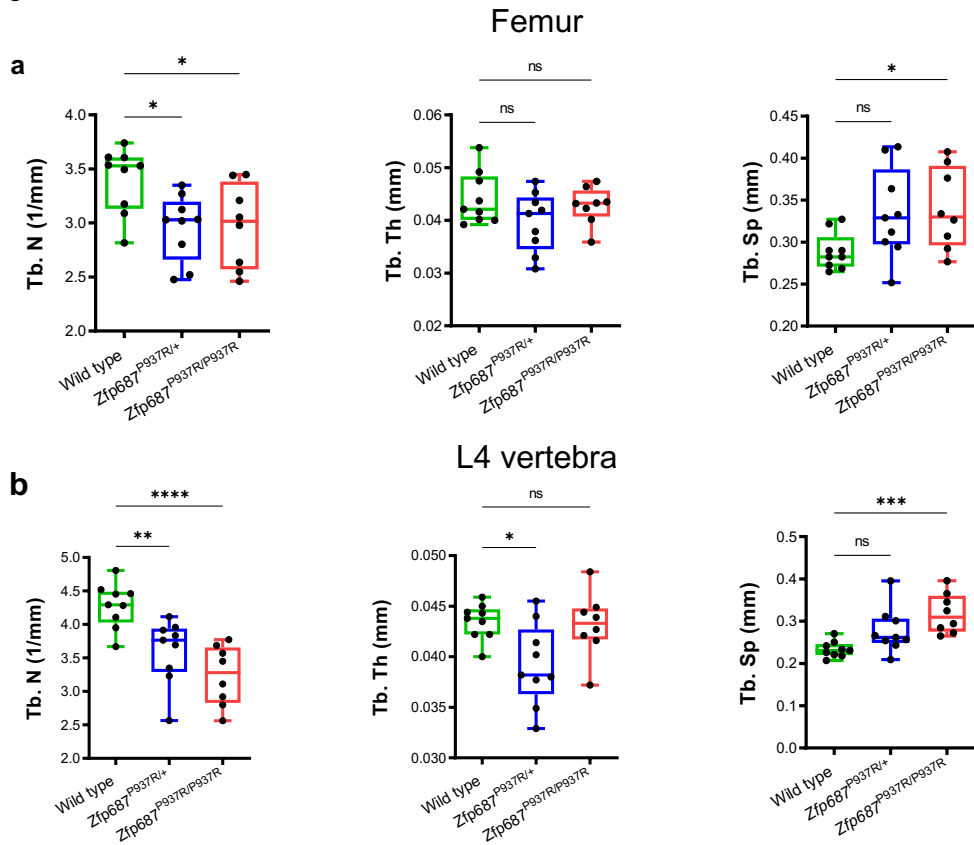

Supplement: Supplementary file 4 — Figure S4 [file 41413_2023_250_MOESM4_ESM.pdf]
