# Supplementary material for: A mutation in the ZNF687 gene that is responsible for the severe form of Paget’s disease of bone causes severely altered bone remodeling and promotes hepatocellular carcinoma onset in a knock-in mouse model
Source: Bone Res. 2023 Mar 14;11:16. doi: 10.1038/s41413-023-00250-3 (PMC10014847; doi:10.1038/s41413-023-00250-3)
Supplement: Supplementary file 6 — Supplementary figure and video legends [file 41413_2023_250_MOESM6_ESM.docx]

**Supplemental Figure S1**: **(a)** Schematic overview of the *Zfp687* targeted allele and the construction of the targeting vector for knock-in. Exons are indicated with boxes; gray indicates untranslated regions, blue indicates coding regions. The c.C2810G mutation has been inserted in exon 6. The long homology arm of the targeting vector includes a genomic region of 5373 bp, from intron 1 to exon 9 (UTR); the short homology arm includes a genomic region of 1501 bp, from exon 9 (UTR) to the intergenic region. The Neo cassette (pGND vector) is flanked by LoxP sites. **(b)** Long sequence alignment of DNA from human *ZNF687* and mouse *Zfp687* using the mVISTA bioinformatics tool. Gene exons are indicated in violet. A threshold of 50% homology is shown.

**Supplemental Figure S2. Three-month-old *Zfp687*^P937R^ mutant mice show no alterations in long bone microstructure.** Trabecular bone parameters, including BV/TV, Tb. N, Tb. Th, Tb. Sp, and Ct. Th, were determined by µCT scan in femur distal epiphysis of 3-month-old wild type (n = 10), *Zfp687*^P937R/+^ (n = 9), and *Zfp687*^P937R/P937R^ (n = 10) mice. BV/TV, bone volume to total volume ratio; Tb. N, trabecular number; Tb. Th, trabecular thickness; Tb. Sp, trabecular separation; Ct. Th, cortical thickness. Data are presented as the median ± s.d. Statistical significance was assessed by one-way ANOVA with Dunnett’s multiple comparison test.

**Supplemental Figure S3. Three-month-old *Zfp687*^P937R^ mutant mice show no alterations in the axial skeleton microstructure.** Trabecular bone parameters, including BV/TV, Tb. N, Tb. Th, and Tb. Sp, were determined by µCT scan in L4 vertebrae of 3-month-old wild type (n = 9), *Zfp687*^P937R/+^ (n = 9), and *Zfp687*^P937R/P937R^ mice (n = 10). Data are presented as the median ± s.d. Statistical significance was assessed by one-way ANOVA with Dunnett’s multiple comparison test.

**Supplemental Figure S4. Histomorphometric analysis of femurs and vertebrae of 8-month-old mice. a)** Trabecular bone parameters, including Tb. N, Tb. Th, and Tb. Sp, were determined by µCT scan in the femoral distal epiphysis of 8-month-old wild-type (n = 9), *Zfp687*^P937R/+^ (n = 9), and *Zfp687*^P937R/P937R^ (n = 8) mice. Data are presented as the median ± s.d. Statistical significance was assessed by one-way ANOVA with Dunnett’s multiple comparison test (*p < 0.05). **b)** Trabecular bone parameters, including Tb. N, Tb. Th, and Tb. Sp, were determined by µCT scan in L4 vertebrae of 8-month-old wild type (n = 9), *Zfp687*^P937R/+^ (n = 9), and *Zfp687*^P937R/P937R^ (n = 8) mice. Data are presented as the median ± s.d. Statistical significance was assessed by one-way ANOVA with Dunnett’s multiple comparison test (*p < 0.05; **p < 0.01; ***p < 0.001; ****p< 0.0001).

**Supplemental Video S1. Compromised and impaired mobility in 16-month-old P937R-mutant mice:** Zfp687^P937R/P937R^ mutant mice showed strongly impaired locomotion, dragging the hindlimbs.
